# On the Torula Cerevisiæ, or Yeast-Plant

**Published:** 1840-04

**Authors:** 


### PART FOURTH.

### Medical Intelligence.

---

### ON THE TORULA CEREVISIÆ, OR YEAST-PLANT.

We are the more induced to notice this curious subject in our present Number, because of its close relationship to the important discoveries of Schleiden and Schwann, detailed in a preceding article (Art. X., p. 495); and we have thought it of sufficient consequence to deserve illustration (see Plate II., Fig. 12, *a*, *b*, *c*). The description of the yeast-globules is derived from a paper in the *Comptes Rendues* (Aug. 20, 1838); and we have ourselves tested its accuracy.

When *yeast* or *ferment* is examined with a microscope, it is seen to consist principally of a number of minute disconnected vesicles, much resembling (except in colour) those of the protococcus (fig. 12, *a*). These, like seeds, may possess a dormant vitality for almost any length of time; their power of growth, when placed in proper circumstances, not being destroyed by exposure to such extremes of temperature as 212° and 76°, or by being entirely desiccated. When these are placed in a saccharine solution, they commence vegetating actively, provided the temperature be sufficiently high. If a fluid in a state of fermentation be examined at short intervals, it is observed that each vesicle puts forth one or more little prolongations or buds (fig. 12, *b*), which in time become new vesicles like their parents; these again perform the same process, so that within a few hours the single vesicles develope themselves into groups of four, five, or six (fig. 12, *c*). This is not the only way, however, in which they multiply: sometimes the vesicles are observed to burst, and to emit their contents; and new individuals originate from the germs thus liberated. This simple form of reproduction has evidently a fundamental correspondence with that which has already been described in regard to the pollen-grain of the flowering-plants, and the spores of the higher cryptogamia; and it exactly corresponds with that of the red snow, which does not, however, *also* increase by buds as this does. By the time that five or six vesicles are found in each group, the fermentation is sufficiently advanced for the purposes of the manufacturer, and he then takes measures to check it. The vegetation of the yeast is then suspended, and the groups of vesicles separate into individuals, the mass of which constitutes the yeast thus largely increased in amount. This discovery was made almost concurrently by three observers, MM. Cagniard-Latour, Schwann, and Kützing.

All fungi appear to have, in a greater or less degree, the power of hastening the decomposition of the organic substances on which they grow, for the purpose, it would seem, of thus procuring for themselves the supply of carbon which other plants derive from the atmosphere. The common *mould*, for example, destroys the palatability of sweet preserves; and the *dry-rot* causes the decay of the timber it infests. Like the rest of its tribe, the simple but important plant we have just described (which has received the appellation of *torula cerevisiæ*) decomposes the organic matter in which it developes itself; and it carries a step further the process which the conversion of the barley into malt by incipient germination had already commenced. This had changed the fecula of the seed into sugar, and an equivalent of carbon was thus liberated. Another equivalent is now abstracted for the use of the fungus, and the sugar is converted into alcohol.

But the wonders of this humble vegetation do not stop here. The same germs which thus develope and multiply themselves in an actively-fermenting fluid may produce a plant of very different aspect at a subsequent time. When malt liquor is becoming stale, a slimy production is found on its surface, which, when microscopically examined, is found to be of a vegetable nature, and to originate almost certainly from some of the vesicles left in the fluid.

In placing a mass of yeast into a fermentable solution, therefore, we sow the germs of myriads of fungi, by the development of which that change is effected in the fluid which it is our object to produce. But, it will be asked, how does fermentation commence in fluids which have not been thus treated? The solution of this question, according to M. Turpin, is to be found in the hypothesis, that the cell-germs (cytoblasts) contained in the vesicles of the malt or other vegetable structure from which the saccharine solution is made, being liberated from their relation with the parent structure, may develope themselves as separate individuals in a lower grade of vegetable existence. In other words, the same germs may produce cells which shall form part of the future barley-plant, or which shall exist as the *torula cerevisiæ,* according to the circumstances of their development. This is certainly a bold hypothesis; but it is one not unsupported by facts. According to M. Turpin, these simple forms of vegetation may take their origin not only from higher plants, but from the results of animal organization. He has observed the *penicillium glaucum* (a common form of mould) to spring from globules of milk; and a form not very dissimilar to arise from the albumen of the egg. Reasons have been lately given for the belief that the globules in the fluids of the living animal body have sufficient independent vitality to multiply themselves by spontaneous rupture and liberation of the contained germs of others; but upon this subject we do not yet feel competent to offer a decided opinion. In the mean time, we think that there is sufficient evidence for the reception of M. Turpin's hypothesis as not improbable; though we ought by no means to build upon it with anything like certainty. It will be seen to fall in with the more general idea which we formerly expressed (Vol. IV., p. 20), that beings of a high degree of organization may, under peculiar circumstances, develope, from various parts of their tissues, forms corresponding with those of inferior organisms.

